# Metabolomic Profiling in Perinatal Asphyxia: A Promising New Field

**DOI:** 10.1155/2015/254076

**Published:** 2015-01-31

**Authors:** Niamh M. Denihan, Geraldine B. Boylan, Deirdre M. Murray

**Affiliations:** Neonatal Brain Research Group, Department of Paediatrics and Child Health and the Irish Centre for Fetal and Neonatal Translational Research (INFANT), University College Cork and Cork University Maternity Hospital, Wilton, Cork, Ireland

## Abstract

Metabolomics, the latest “omic” technology, is defined as the comprehensive study of all low molecular weight biochemicals, “metabolites” present in an organism. As a systems biology approach, metabolomics has huge potential to progress our understanding of perinatal asphyxia and neonatal hypoxic-ischaemic encephalopathy, by uniquely detecting rapid biochemical pathway alterations in response to the hypoxic environment. The study of metabolomic biomarkers in the immediate neonatal period is not a trivial task and requires a number of specific considerations, unique to this disease and population. Recruiting a clearly defined cohort requires standardised multicentre recruitment with broad inclusion criteria and the participation of a range of multidisciplinary staff. Minimally invasive biospecimen collection is a priority for biomarker discovery. Umbilical cord blood presents an ideal medium as large volumes can be easily extracted and stored and the sample is not confounded by postnatal disease progression. Pristine biobanking and phenotyping are essential to ensure the validity of metabolomic findings. This paper provides an overview of the current state of the art in the field of metabolomics in perinatal asphyxia and neonatal hypoxic-ischaemic encephalopathy. We detail the considerations required to ensure high quality sampling and analysis, to support scientific progression in this important field.

## 1. Introduction

Over the past decade, the search for useful biomarkers to accurately predict injury severity in perinatal asphyxia and hypoxic-ischaemic encephalopathy (HIE) has become a growing area of interest in neonatal research. Despite the potential of many promising markers, few studies have been validated or have been able to translate their findings into clinical practice [1]. Metabolomics provides a unique snapshot of human metabolism, reflecting the body's phenotype and ultimately its state of health at a point in time. The ability of metabolomics to measure rapid alterations in metabolism has potential for novel developments in neonatal medicine [2].

Perinatal asphyxia can arise from a number of antepartum and intrapartum risk factors including severe maternal anaemia or hypertension, traumatic birth, or interruption to the umbilical cord circulation during labour which results in cessation of respiratory gas exchange to the fetus. It remains a persistent worldwide problem occurring in 20 per 1000 term live births of which 2-3 per 1000 will develop a subsequent encephalopathy. In the developing world this figure is dramatically higher at 5–10 per 1000 [3, 4]. HIE is an evolving process and is the culmination of a “two step” injury. An initial cerebral hypoxic and/or ischemic insult is characterised by a switch to anaerobic metabolism and accumulating reactive metabolites resulting in severe energy depletion. While neuronal cells display a transient or “latent” phase recovery from the initial cerebral energy hit, this is followed 6–48 hours later by a secondary injury due to a wave of further energy failure; the formation of free radicals, proteases, and caspases which lead to permanent neuronal cell damage through necrosis and apoptosis [5]. Serious consequences of HIE include motor and cognitive disabilities [6] along with visual loss, hearing loss, behavioural difficulties, or long-term epilepsy in up to 30% of infants [7, 8].

The advent of therapeutic hypothermia has for the first time allowed us to intervene and alter the course of this disease with improved rates of intact survival [9]. Unfortunately, to be effective hypothermia must be initiated within the first 6 hours of birth following the “latent” phase [10]. This has heightened the need for both early diagnosis of HIE and a better understanding of its initial pathophysiological mechanisms. Current markers used to assess the severity of the injury in routine clinical practice are unreliable and do not accurately predict long-term outcome [11]. A quick, cost effective, reproducible, noninvasive, and user independent method for quantifying HIE severity and likely prognosis is urgently needed [12].

The underlying metabolic mechanisms of HIE are poorly understood, particularly the complex interactions of individual metabolic pathways. Current understanding of the injury indicates that hypoxia-ischaemia triggers a biochemical cascade of events [13], characterised by reduced oxygen and glucose which alters neuronal and glia homeostasis. Neuronal supply of high energy metabolites such as adenosine triphosphate (ATP) diminishes, and the sodium/potassium (Na^+^/K^+^) ion pumps dependant on ATP being to fail. This leads to mitochondrial membrane depolarization followed by an accumulation of sodium and calcium in parallel with the depletion of potassium in cells, which causes cytotoxic edema [14]. The extracellular buildup of excitatory amino acids coupled with intracellular Ca^2+^ entry attenuates the neuronal cell swelling [15]. Moreover, hypoxia-ischaemia initiates oxidative stress reactions as the reduction in molecular oxygen is coupled with production of reactive oxygen species, reactive nitrogen species, and impaired oxidative phosphorylation [16]. All contributed to, by the switch to anaerobic metabolism and resulting metabolic acidosis described by the impaired removal of metabolic and respiratory by-products in particular, lactate and pyruvate [17, 18]. This complete failure of cerebral mitochondrial activity can have profound global metabolic effects and leads ultimately to irreversible cell death.

The metabolic response to hypoxia remains unclear, as does its role in alleviating or attenuating the injury sustained. We can no longer rely on a reductionist approach which has traditionally focused on small numbers or individual metabolites in isolation, when large scale disruption of multiple pathways is likely to occur. An integrative systems biology approach is needed to understand the behaviour of all biological components in a system, particularly their mechanistic interactions. Metabolomics combines high throughput analysis with theory, bioinformatics, and computational statistics [19, 20]. Therefore it has the potential to elucidate disease mechanisms in perinatal asphyxia by describing qualitatively or quantitatively the activation and interaction of metabolic pathways and ultimately depicting an infant's phenotype [21]. A unique metabolomic fingerprint of hypoxia-ischaemia could be used to indicate injury severity and predict response to treatment and potential neurodevelopmental outcome. The study of metabolomic biomarkers in the immediate neonatal period requires a number of specific considerations, unique to this disease and population. In this paper we outline current research in this exciting field and the unique challenges posed by the study of metabolomics in perinatal asphyxia and HIE.

## 2. Early Work in the Field

Huang et al. (1999) began this search by examining the early urinary ratio of lactate to creatinine in asphyxiated neonates, using proton nuclear magnetic resonance (^1^H-NMR) spectroscopy. They defined a lactate to creatinine ratio value of 0.64 or higher, within 6 hours of birth, to be highly specific (100%) and sensitive (94%) for predicting the development of HIE (*n* = 16). Furthermore, the mean ratio was significantly higher in infants who had an adverse outcome at one year of life [22]. A urinary metabolomic profile of infants with clinical evidence of severe asphyxia has also been described using gas chromatography-mass spectrometry (GC-MS), which linked four energy and four oxidative stress metabolites to good and poor infant outcome, respectively [23]. While this initial work proved very promising, the severity of injury, timing of collection, and outcome of the infants were poorly defined.

## 3. Animal Models

Several studies have employed animal models of asphyxia/hypoxia to study metabolomic alterations and a number of putative metabolomic fingerprints have been proposed. The accumulation and delayed recovery of Krebs cycle intermediates (e.g., fumarate, succinate, malate, and alpha ketoglutarate) have been described in a range of models [24–27]. These energy metabolites are a product of the shift toward anaerobic conditions, illustrated by the accumulation of lactate [22, 28, 29], and mitochondrial dysfunction [30] leading to disturbance of the Krebs cycle. Due to reduced ATP availability, these metabolites cannot enter the respiratory chain for energy production and have been suggested as possible cofactors in lactate formation [24].

Altered amino acid profiles were shown in a number of animal studies [26, 31, 32]. Amino acid ratios of alanine to branched-chain amino acids (BCAA) and of glycine to BCAA, in combination with the energy metabolites succinate and propionyl-*L*-carnitine, were shown to highly correlate (*R* ^2^ = 0.96) with the duration of hypoxia in a piglet model [24]. BCAA act as alternate energy sources for the brain and muscles [32, 33]. Their marked presence in metabolomic studies has been confirmed also in asphyxiated neonates [15] and may illustrate their mobilisation under conditions of reduced ATP production. However valine, a BCAA known to increase during excitotoxicity, has been described as contributing to hypoxia-ischaemia facilitating the return of nitrogen to astrocytes [34].

Disturbance of the cell membrane, perhaps through the release of oxygen free radicals which cause the oxidative degradation of lipids and membrane dysfunction, has been illustrated by the marked elevation of arachidonic acid, a constituent of the phospholipid bilayer [25]. Metabolomic examination of neuronal tissue revealed three differentiating metabolites (CDP-choline, choline and acetylcholine) which participate in glycerophospholipid metabolism [35], the main component of cell membranes. Lastly, urinary metabolites which are reflective of kidney function have displayed potential in predicting death in newborn piglets [30], reflecting the systemic organ failure caused by severe hypoxia.

Overall, animal models have made important contributions to the field of neonatal medicine. Most notably, translational research of this nature provided the evidence base to administer therapeutic hypothermia to neonates with HIE, which is now standard of care [36]. Clinically a hypoxic-ischaemic insult is unpredictable, usually occurring in the perinatal period, but the exact timing and duration are often difficult to estimate. Animal models provide the ability to control the timing of the injury, allowing for the timed detection of metabolite alteration and elucidation of disease mechanisms. Genetic and environmental variability can be tightly regulated ensuring the phenotype is representative of the injury, unlike the dynamic inter- and intraindividual variability in humans, which demands large sample numbers to statistically control [37]. They also overcome ethical issues, for example, using a nontreatment or normothermia control group.

Despite these obvious advantages, animal models may not capture the complex organ interactions and multiple comorbidities, especially neurodevelopmental ones, associated with HIE. Many models exist but there is no consensus on which best mimics the pathophysiological and clinical condition [38]. As a result, animal studies have varied in terms of species, outcome measures, method of asphyxial injury, biospecimen sampled, and metabolomic approach, presented in Table 1.

Apart from Beckstrom et al. [25], animal models in this field have generally assessed neonatal but not the clinically occurring in utero or perinatal asphyxia. Newborn piglet models have induced systemic hypoxia by lowering the fraction of inspired oxygen (FiO_2_) to between 0.06 and 0.13; however, this fails to inflict the clinically concurrent ischaemia associated with HIE. A rodent model by Vannucci has classically combined unilateral carotid artery ligation with 8% oxygen to mimic perinatal asphyxia [39]. This model was used by Liu et al., to assess brain tissue metabolites in postnatal day 7 mice [34], which were originally considered to be equivalent to a 32–36 week gestation human infant [40]. Choosing an optimal animal model whose brain is equivalent to that of a term newborn infant remains contentious [41].

In the aforementioned studies, animals are usually sacrificed soon after recovery for histological examinations; no definitive diagnosis of HIE is provided and the extent of the brain injury is not assessed. Combining imaging and outcome studies with animal models is becoming increasingly important to assess the phenotype of newborn injury. Larger animals can be recovered after injury and their physiological and neurobehavioral responses assessed; for example, magnetic resonance imaging (MRI) and electroencephalogram (EEG) can identify injury severity and seizure activity [42, 43]. Yet, subtle long-term deficits in memory or processing speed remain difficult to measure.

Metabolites generally reflect the genetic divergence between species. However, distinct concentration differences were evident when human metabolomes of the prefrontal cortex, primary visual cortex, cerebellar cortex, kidney cortex, and thigh skeletal muscle were compared to those of chimpanzees, macaque monkeys, and mice [44]. This study reported that the metabolites of human prefrontal cortex undergo a 4-fold divergence from their expected evolutionary lineage. Perhaps related to cognitive functions unique to humans, indicating the advanced human metabolome may not be directly comparable to those of animals.

Finally, ethical considerations in animal research to “replace, reduce and refine” have resulted in study cohorts being relatively small making it difficult to extrapolate meaningful results. For example, Murgia et al. assessed the association between metabolite changes and recovery time after hypoxia in 10 piglet subjects but commented on the distinct basal interindividual metabolomic differences among subjects [31].

Though animal experiments are providing valuable information in this developing field, the varying cerebral maturation and metabolism of species mean that no one model reflects the human condition [45]. Many early metabolomic investigations have failed to comply with the minimum reporting standards for metabolomic analysis which aim to enable the interrogation, replication, and comparison of data [46]. This is impinging the translation of animal findings to the human scenario. To progress metabolomic animal experimentation toward clinical benefit, parallel validation studies in cohorts of human neonates are essential [47].

## 4. Translation of Animal Research to Human Neonatal Studies

### 4.1. Study Population

A major limitation in neonatal biomarker studies is the difficulty of recruiting a sufficient and appropriate study population. Disease prevalence of HIE is low (0.3%) making it difficult to collect representative high quality samples in adequate numbers suitable for metabolomic biomarker discovery. Therefore, even in large maternity hospitals with 5–10 thousand deliveries per year and with full recruitment, only 10–20 cases of moderate-severe HIE and 20 cases of mild HIE per annum would be expected. In a study that recruited 256 newborns from three separate hospitals, only 11 were cases of severe asphyxia (8 HIE and 3 perinatal death) compared to 216 healthy controls (16 excluded) [23]. Human neonatal biomarker studies clearly require standardised recruitment and clear case definition across multiple centres.

### 4.2. Case Definition and Recruitment

To ensure a biomarker is in fact detecting hypoxic-ischaemic injury we must rule out the many other causes of a neurologically depressed neonate at birth. Infants with metabolic encephalopathies, sepsis, genetic abnormalities, or neuronal migration disorders can all present with low Apgar scores and poor tone in the first days of life [48, 49]. To progress biomarker research we need to surpass the “default diagnosis” of neonatal encephalopathy [48] and carefully define our population using clear recruitment criteria.

Standardised recruitment poses its own challenges; firstly the clinical grading of HIE can vary between centres. To standardise the grade of HIE, EEG, and amplitude integrated EEG monitoring from as soon as possible after birth has proved very useful [50, 51]. A definitive diagnosis of HIE is often not available in the immediate postnatal period, and so the opportunity to collect early biosamples may have passed. One solution is to collect samples from all infants “at risk” of HIE, using study inclusion criteria encompassing broad signs of birth asphyxia, for example, ≥36 weeks of gestational age, Apgar score ≤ 6 at 5 minutes, cord blood pH < 7.1, or requiring intubation or CPR at birth [22]. Inevitably infants with perinatal asphyxia but without HIE will be recruited; this must be favoured over omitting infants who progress to display more severe clinical signs later in the postnatal period.

### 4.3. Sample and Data Collection

The highly unpredictable nature of HIE requires a regimented and multidisciplinary approach to identify participants and collect samples 24 hours a day, 365 days of the year. The acute clinical setting demands the participation of medical, nursing, midwifery, and research staff from both neonatal and maternity units. A biomarker suitable for early diagnosis of HIE must be detectable soon after birth; therefore the specimens used in the discovery phase should be collected, processed, and biobanked, from every “at risk” infant, within 3 hours of birth. This short window may mean the reliance on labour ward staff to identify study candidates, draw a sample, and notify the research team immediately after delivery. Informed parental consent can be sought after delivery when clinically appropriate, due to the inability to identify cases prior to birth. Control infants can be simultaneously recruited by consenting antenatally.

The demographic variability of the HIE population will itself account for metabolomic differences; for example, gestational age creates a distinct metabolomic profile [52], as do mode of delivery [53] and intrauterine growth [54]. Other possible confounding factors include gender, ethnicity, maternal BMI, and the length of time the blood sample has spent in the freezer. Matching cases 1 : 1 with controls is vital to achieve adequate power with smaller numbers [55], while incorporating demographic, physiological, and lifestyle factors into a matched cohort will help to eliminate their potential discriminatory bias on the metabolomic model [37].

### 4.4. Specimen Biobanking

High quality biospecimens are essential for metabolomic research to ensure the sample is a representative snapshot of the metabolome at the time of collection [19]. As evident from the animal research discussed, many different types of biological fluids and tissues can be analysed. Each biospecimen will give a unique fingerprint of biochemical changes and complex interactions in a particular region, depending on the physiological or environmental perturbation. In maternal-fetal metabolomic investigations a range of biospecimens have already been employed including plasma, urine, amniotic fluid, placental tissue, vaginal secretions, and cord blood [56]. It is important to note that no single specimen will reflect the total metabolomic disturbance of an organism; therefore one must be carefully chosen based on availability, suitability to achieve the study aims, and level of invasiveness.

For infants with perinatal asphyxia and HIE, postnatal specimen collection is difficult and can easily be confounded by progression of disease, postnatal interventions, or timing of collection. The average 3.5 kg infant will have only 280 mL of blood. Ethical guidelines limit research sampling to 1% of circulating blood volume per 24 hours, giving an upper limit of 3 mL for postnatal sampling, or approximately 1–1.3 mL of serum or plasma. This limited availability of blood for metabolomic analysis leaves little room for error or internal validation.

In our opinion, the metabolomic analysis of umbilical cord blood (UCB) offers the unique potential to identify the severity of cellular hypoxia and resulting damage. It is suited to biomarker discovery as large volumes (20 mL) can be noninvasively extracted from the placenta by clamping the cord at each end, allowing for whole blood, serum, and plasma to be biobanked. A mixed arterial/venous cord sample simplifies collection and user friendly testing in the acute setting. Ideally, UCB will be drawn from the placenta within 20 minutes of birth and immediately stored at 4°C before processing in a sterile environment, to quench enzymatic activity and ensure subsequent biological observations are accurate. Biobank freezers should be housed in a facility containing temperature control, ventilation, and generator backup power and monitored 24 hours a day to ensure no metabolic degradation has occurred. Biobanking samples in microaliquot volumes that are sufficient for one metabolomic experiment will eliminate freeze thaw cycles which alter metabolites [19].

### 4.5. Validation

Promising metabolomic biomarkers require manifold validation. Before a metabolomic biomarker can reach the clinic it needs to progress through a pipeline of biomarker discovery, study validation, and cohort validation [19]. Typically biomarker discovery will use an untargeted metabolomic method whereby analytical data is detected for a large range of metabolites (hundreds or thousands) simultaneously. This method is hypothesis generating and no* a priori* knowledge of the metabolites of interest is needed. However, when using high throughput and highly sensitive untargeted methods such as mass spectrometry, precision and accuracy are reduced and the potential for a random chance discovery is especially high, even when statistical corrections are applied. Therefore, careful validation of the study design, conduct, and interpretation is needed to ensure findings are reproducible and can progress to the more costly cohort validation.

Study validation requires prospective planning and the inclusion of quality assurance (QA) and quality control (QC) systems is essential in limiting preanalytical errors that produce false discoveries [57]. QA measures (planned process activities) can be embedded into standard operating procedures (SOPs), which provide step-by-step procedures for sample handling, processing, and curation, helping to minimise variability in staff and across sites. Randomisation of sample preparation and analysis, within and between sample batches, along with blinded analysis and interpretation will aid validation of the analytical methods and rule out bias based findings [46].

To confirm data quality, QC samples have become a particularly important strand of validation in metabolomics. Scattered throughout the analytical batch, they facilitate the assessment of metabolite recovery after extraction, correction of instrument drift, analyst variation, and data cleaning [37]. Pooling serum from each infant into a single QC sample is optimal to guarantee it represents the population and to ensure the presence of differentiating metabolite signals is genuine.

Finally, a cross validation system can be incorporated by analysing one microaliquot of each infant sample on independent metabolomic platforms. Although the sensitivity of each platform allows for the identification of specific metabolites classes, some do overlap and can help confirm experimental findings. For example, using the same infant cohort several amino acids (methionine, alanine, isoleucine, phenylalanine, valine, and leucine) were identified as being significantly different between HIE and matched controls using both ^1^H-NMR and direct infusion mass spectrometry (DIMS) platforms separately [58, 59]. Statistical techniques also require cross validation to avoid false discoveries, as powerful machine learning techniques can easily overmodel data that is indeed artefact [43].

Cohort validation is performed using a larger, independent neonatal cohort and should focus on a short list (<20) of valid and reproducible candidate biomarkers. Here, a targeted metabolomic approach is employed to specifically examine the candidate metabolites using distinct extraction methods, MS/MS, and isotopic internal standards for absolute identification and quantification. Quantitative measurements have the added advantage of being easier to validate across centres and translate more smoothly into a diagnostic test. Unfortunately thus far, no metabolomic findings in perinatal asphyxia and HIE have made the transition to cohort validation, due to the challenges already described.

### 4.6. Outcome Measures

The potential of early robust metabolomic biomarkers in neonatal HIE is manifold:

- during labour: allowing the identification of infants with significant perinatal asphyxia, at risk of HIE when obstetric intervention is required;
- immediately postnatally: to identify those infants who are likely to progress to moderate-severe encephalopathy and may benefit from neuroprotective intervention;
- postnatally: to assess response to therapy and predict long-term outcome.

Once again, to accurately assess our ability to predict outcome, standardised methods are required. Early outcome consists of grade of encephalopathy and survival. Examination at discharge is helpful but has low specificity for long-term deficits. More subtle difficulties in learning and memory may not become apparent for many years but provide the bulk of the societal burden following neonatal HIE. Learning impairment, neuropsychological deficits, and behavioural difficulties are seen in over 40% of surviving children at school age [60].

Therefore, long years of recruitment need to be superseded by years of careful follow-up to progress this rapidly evolving field. Retaining participants for long-term follow-up studies can be difficult and continued communication with parents is essential [61].

## 5. Metabolomic Profiling of Umbilical Cord Blood

Despite these challenges, some progress has been made. The biochemical derangement in UCB after a neonatal hypoxic-ischaemic injury has recently been reported. A quantitative DIMS approach showed significant alterations between groups (HIE versus matched control, perinatal asphyxia versus matched control) in 29 serum metabolites from three distinct classes: amino acids, acylcarnitines, and glycerophospholipids [59]. Energy production depends on the conversion of amino acids to glucose and ketone bodies or their role as precursor metabolites to the Krebs cycle [33]. Disruption of the Krebs cycle has been shown to increase its intermediates [24], which may account for the elevated precursor amino acid concentrations. When functioning normally, acylcarnitines transport long chain fatty acids across the mitochondria via a carnitine shuttle. Acylcarnitine levels were increased in neonates with both perinatal asphyxia and HIE relative to matched controls, suggesting a defect in fatty acid oxidation, resulting in their accumulation and release into circulation [62]. This metabolite model could differentiate between HIE and controls with an AUC of 0.93 and demonstrated the potential to distinguish between injury severities.

A 1D ^1^H-NMR spectroscopy method was used to specifically characterise primary energy metabolites, finding significant changes in 18 UCB metabolites from infants with either perinatal asphyxia or HIE [58]. A metabolic profile of severe HIE was illustrated by the mean fold increase of succinate and glycerol and decrease of ketones, acetone, and 3-hydroxybutyrate. Ketones act as an alternate energy source for the infant brain and, in severe HIE, ketone depletion suggests that the magnitude or duration of hypoxia-ischaemia has depleted these stores and reduced critical ATP generation [63, 64]. Mild hypoxia has previously shown no elevation in 3-hydroxybutyrate in contrast with severe hypoxia [32]. The presence of acetoacetate and succinate post hypoxia has been reported in animal models [31]. Acetoacetate is integral to ketogenesis whereby it is decarboxylated to acetone or reduced to 3-hydroxybutyrate, which in turn can be transformed to acetyl-CoA for energy production and hence the release of succinate. Many animal models have described the alteration of succinate but not its association with HIE severity. Succinate was found to increase 8-fold in severe HIE compared to 2-fold in mild/moderate HIE, suggesting it plays an important role in disease mechanisms. It potentially promotes a shift to anaerobic metabolism by stabilising hypoxia-inducible factor 1*α* (HIF1 *α*), an important component in the hypoxic stress response. Whilst these results are promising and are supported by animal data, they have yet to be validated in an additional prospective neonatal cohort. This is the next challenge in order to progress the field towards clinically useful biomarkers.

## 6. Conclusion

In summary, a number of putative metabolite fingerprints persist throughout the literature discussed. Metabolomic data thus far is consistent with the perturbation of energy metabolism and mitochondrial injury in hypoxia-ischaemia. In particular both human and animal work have described the alteration of Krebs cycle metabolites [23–27, 58], amino acids [24, 26, 31, 58, 59], and constituents of the cell membrane [25, 35, 59]. These metabolic changes are consistent with the current understanding of injury mechanisms, evidence of strong biological plausibility.

Metabolomic markers have the potential for translation into diagnostic, predictive, or screening tests in perinatal research. The immediate goal is to produce an innovative panel of metabolites to diagnose the degree of HIE quickly, reliably, and cost effectively at the cot-side. Emerging technologies are facilitating the point of care measurement of metabolites, with software algorithms allowing the incorporation of clinical data with metabolite measurements using only a few microliters of blood [65].

Tailored management systems could arise from the early detection of infants likely to have poorer outcomes, leading to targeted intervention or novel therapies for those most at risk. Large multicentre metabolomic biomarker studies are now required to verify these early putative findings. QA and QC systems are important to ensure specimens have been biobanked and phenotyped precisely across multiple sites and are suitable for translational research.

We wish to encourage researchers in the field to validate their finding in separate neonatal cohorts. A community focused programme of research is urgently needed to ultimately develop neonatal bedside tests with significant and real health benefits in this vulnerable population.

## Figures and Tables

**Table 1:** Summary of metabolomic studies in animal models of hypoxia/asphyxia.

Author, year	Ref	Study population	Specimen	Analytical platform	Metabolite alteration	Key finding
Fanos et al., 2014	[66]	Newborn piglets	Urine	^ 1^H-NMR	15 metabolites	Resuscitation with 21% O_2_ improves cellular recovery

Arduini et al., 2014	[29]	Newborn piglets (12–36 hours old)	Retinal & choroid tissue	UPLC-MS/MS	Lactate	↓mitochondrial metabolites, anaerobic shift

Murgia et al., 2013	[31]	Newborn piglets	Urine	^ 1^H-NMR	n-Phenylacetylglycine, alanine, acetoacetate, methanol, glucose, sarcosine, succinate, dimethylamine	Reoxygenation speed alters metabolite profile

Solberg et al., 2013	[35]	Newborn piglets (12–36 hours old)	Retinal tissue	UPLC-QTOFMS UPLC-MS/MS	CDP-choline, acetylcholine, and choline	Phospholipid synthesis involvement and neuroprotective metabolites

Skappak et al., 2013	[26]	Newborn piglets (1–3 days old)	Urine	^ 1^H-NMR	Amino acids, energy metabolites, 1-methylnicotinamide & betaine	Urinary metabolites identify hypoxia

Liu et al., 2013	[34]	Mice (postnatal day 7)	Brain tissue	^ 1^H-NMR	Energy metabolites, ATP-ADP, valine, phosphocholine, lactate, glucose, and glutamate	Hypothermia treatment alters metabolites

Beckstrom et al., 2011	[25]	Macaques	Plasma (5 min after birth)	2D GC/MS	10 metabolites	Krebs cycle metabolites altered and cell membrane disturbance

Solberg et al., 2010	[24]	Newborn piglets	Plasma	LC-MS/MS FIA-MS/MS	Alanine/BCAA, glycine/BCAA (correlation *R* ^2^ = 0.96), and carnitines	↓Krebs cycle intermediates & delayed recovery with 100% O_2_ resuscitation

Atzori et al., 2010	[30]	Newborn piglets	Urine	^ 1^H-NMR	Creatinine, methylguanidine, hydroxyisobutyric acid, urea, and malonate	Prediction of death

van Cappellen van Walsum et al., 2001	[32]	Fetal lambs	CSF	^ 1^H-NMR	Amino acids, BCAA, lactic acid, and hypoxanthine	Prolonged mild hypoxia leads to energy degradation

Ref: reference; ^1^H-NMR: proton nuclear magnetic resonance (^1^H-NMR) spectroscopy; UPLC-MS: ultraperformance liquid chromatography-quadrupole time of flight mass spectrometry; UPLC-MS/MS: ultraperformance liquid chromatography-tandem mass spectrometry; GC/MS: two-dimensional gas chromatography-mass spectrometry; LC-MS/MS: liquid chromatography-tandem mass spectrometry; FIA-MS/MS: flow injection analysis-tandem mass spectrometry; BCAA: branched-chain amino acids; CSF: cerebrospinal fluid.
